# Morphometry of the sural nerve in diabetic neuropathy: a systematic review

**DOI:** 10.1007/s40477-024-00875-y

**Published:** 2024-03-08

**Authors:** Zhang Ludi, Matthias Yi Quan Liau, Bryan Song Jun Yong, Amanda Sze Yen Auyong, Quah Hui Ting Lynette, Samuel Jianjie Yeo, Khin Swee Elizabeth Tan, Sreenivasulu Reddy Mogali, Ramya Chandrasekaran, Vivek Perumal, Ranganath Vallabhajosyula

**Affiliations:** Lee Kong Chian School of Medicine, Nanyang Technological University Singapore, Singapore, 308232 Singapore

**Keywords:** Morphometry, Ultrasonography, Sural nerve, Diabetic polyneuropathy, Diabetes mellitus

## Abstract

**Purpose:**

The aim of this systematic review is to evaluate the usefulness of sural nerve ultrasonography in diagnosing diabetes mellitus (DM) and diabetic polyneuropathy (DPN), the latter of which is a common long-term complication for diabetic patients that frequently involves the sural nerve.

**Methodology:**

A meta-analysis of the cross-sectional areas (CSAs) of sural nerves in healthy individuals and patients with diabetes mellitus based on a total of 32 ultrasonographic-based studies from 2015 to 2023 was performed. Sub-analyses were performed for factors such as geographical location and measurement site.

**Results:**

The meta-analysis showed that the mean CSA of the sural nerve was significantly larger in DM patients with DPN only compared to healthy individuals across all regions and when pooled together. An age-dependent increase in the CSA of healthy sural nerves is apparent when comparing the paediatric population with adults.

**Conclusion:**

Sural nerve ultrasonography can distinguish diabetic adults with DPN from healthy adults based on cross-sectional area measurement. Future studies are needed to clarify the relationships between other parameters, such as body metrics and age, with sural nerve CSAs. Cut-offs for DPN likely need to be specific for different geographical regions.

## Introduction

As of 2021, it is estimated that 1 in 10 adults live with diabetes mellitus (DM) [1]. This highly prevalent disease includes complications that significantly impair one’s quality of living, such as diabetic polyneuropathy (DPN), which is estimated to eventually affect up to 50% of diabetic patients [2]. DPN involves peripheral nerve damage from a variety of molecular mechanisms driven by inflammation, oxidative stress, and ischaemia, resulting in nerve dysfunction that can precipitate further complications with high morbidities, such as foot ulceration, gangrene, and Charcot’s joint. The onset of DPN is gradual, with diagnosis of DPN occurring years after the point of diagnosis of DM for many patients [3]. Hence, new methods of detecting early pathological developments related to DPN could help improve the prognosis of DM and DPN patients.

The involvement of the sural nerve in DPN is common, possibly due to length-dependent exposure to chronic hyperglycaemia and cardiovascular risk covariates that induce metabolic and micro vessel alterations [4–7]. Separately, DPN severity strongly correlates with the severity and duration of diabetes mellitus [8]. Therefore, DPN has traditionally been diagnosed through clinical symptoms and signs and confirmed objectively by abnormalities on nerve conduction studies (NCS) of such nerves [9].

While NCS as an objective measure of the nervous system remains the most reliable evaluation method, NCS provides limited information about the morphology of nerves and their surrounding structures [10, 11]. Further, nerve action potentials are often unexcitable in patients with more advanced DPN [11]. On the other hand, peripheral nerve ultrasonography is a cheap and non-invasive tool able to examine whole nerve courses. It is also widely accessible across most hospitals and may be a potential tool to evaluate peripheral neuropathies via imaging and measurement of nerve fibres.

Given the potential of peripheral nerve ultrasonography as a diagnostic tool of DPN, measurable parameters for the normal morphology of the SN and morphological changes in patients with DPN need to be established to reliably discriminate between the different grades of DPN severity. Currently, the cross-sectional area (CSA) of a peripheral nerve is the most accepted parameter as a reference for the size of a specific nerve [12]. Notably, increased CSAs at non-compressive nerve sites have been observed in DPN patients in some preliminary studies [13–15]. Taken together with the frequent involvement of the SN in DPN, these studies suggest that ultrasonography of the sural nerve may be employed as a diagnostic tool for DPN based on cut-offs of their CSAs.

Therefore, this systematic review aims to collate and perform a meta-analysis of the CSAs of sural nerves in normal healthy individuals and DM patients based on the ultrasonographic-based studies available in current literature.

### Anatomy of the sural nerve

The sural nerve is characterized by extensive anatomical and topographical variability, as demonstrated through both cadaveric and ultrasonographic modalities [16]. Classification of the sural nerve has also changed significantly over time, from originally three patterns to the recent six distinct variants with two more additional subgroups [16, 17]. Recent reviews also show that ultrasonographic and cadaveric studies tend to pick up different types of sural nerve formations at different frequencies, possibly from the shifting of anatomical structures during cadaveric dissection [16, 18].

**Fig. 1:**
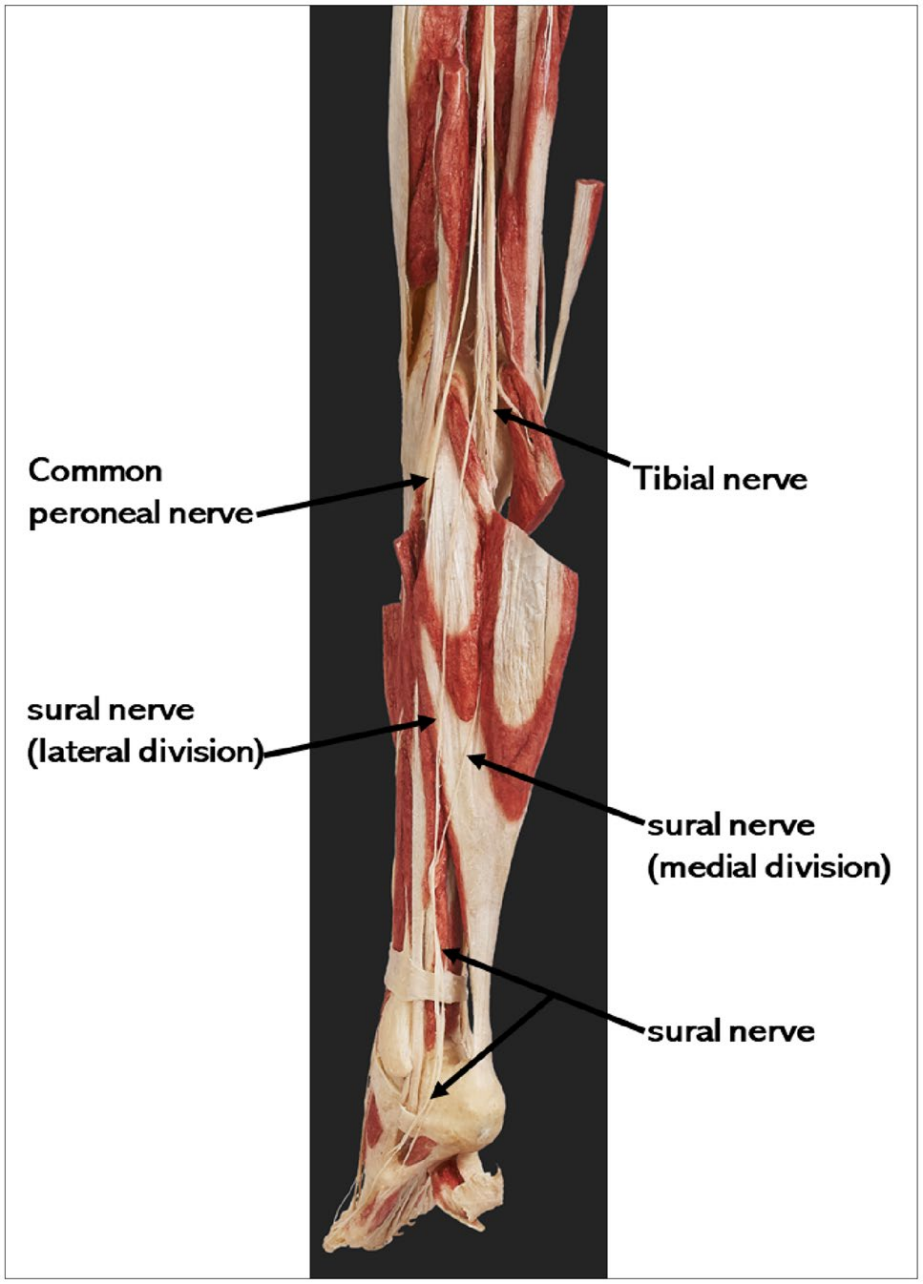
Plastinated specimen image showing the sural nerve. Image source: collection of plastinated specimens at anatomy learning centre (ALC), Lee Kong Chian School of Medicine Singapore

Despite heterogeneity in classification and terminology, the sural nerve is typically a branch of the tibial nerve that descends between the heads of the gastrocnemius and that is joined by the sural communicating nerve, a nerve branch arising from the common fibular nerve. Alternatively, some authors describe the main trunk arising from the tibial nerve as the medial sural cutaneous nerve and the sural communicating nerve from common fibular nerve as the lateral sural cutaneous nerve [19–21]. An anatomical figure of the sural nerve is provided in Fig. 1.

The sural nerve descends in a highly variable course along the Achilles tendon while in close relation to the small saphenous vein from the apex of the calf until it passes into the foot by running 1.5 cm postero–inferiorly to the lateral malleolus. Along this course, it supplies sensory innervation to the skin on the postero-lateral lower third of the leg and continues as the lateral dorsal cutaneous nerve to supply the lateral aspect of the foot and fifth toe [19, 22]. Although there exist variations in its course and distribution, the SN is easily accessible and hence, frequently used for peripheral nerve biopsies and harvesting for nerve grafting [16, 23].

## Methodology

### Search strategy and inclusion criteria

A systematic search was conducted in five major databases (EMBASE, Cochrane, Scopus, PubMed, and Web of Science). The search terms used a combination of keywords such as “sural nerve, ” “morphometry, ” “ultrasonography, ” and “cross-sectional area.” The search was conducted from 1 January 2015 to 23 June 2023. The inclusion criteria included research articles which (1) reported sural nerve CSA measured using ultrasonography for healthy and/or diabetic populations and (2) included full text articles reported only in the English language. Exclusion criteria included (1) single case reports, conference abstracts and systematic reviews, (2) articles where data of healthy and/or diabetic populations were mixed with data of other polyneuropathic populations for analysis and (3) articles with insufficiently clear methodological and/or data reporting, as determined via discussion between authors based on the Anatomical Quality Assessment (AQUA) tool. A systematic screening of literature was performed by at least two members of a team of six independent investigators based on the titles and abstracts. Studies that reported relevant and extractable anatomical data on sural nerve were screened. The search results from various databases were exported to Covidence, a systematic review management software (Veritas Health Innovation, Melbourne, Australia) and subsequently duplications records were excluded. The selection process was compiled and documented as per Preferred Reporting Items for Systematic reviews (PRISMA) guidelines.

### Data extraction

The empirical data from the included articles were extracted and any discrepancies regarding inclusion of studies were resolved by detailed discussion among the investigators. The extracted data were rounded off to three significant figures and were recorded in Microsoft Excel 2010 spreadsheet (Microsoft Corp., Redmond, WA) for further assessment. All data related to the type of study methodology, geographic location, age, measurement side (left or right), and morphometric measurements were extracted and recorded. Quantitative descriptives such as mean CSA, number of SNs and standard deviation (SD) for healthy and diabetic populations were extracted. Some studies did not specifically report SD, instead providing interquartile ranges (IQRs) and/or upper- and lower-bounds. An estimate of standard deviation was calculated manually using these values reported from the original studies as per Cochrane guidelines [24]. As per Cochrane guidelines, studies which included range data only were not included in the meta-analysis due to an inability to accurately estimate standard deviation [24].

### Statistical data analysis

The data from the included papers were grouped as continuous variables and meta-analysis were performed using Open Meta-Analyst software using R console (CEBM, Brown University). A continuous random-effects model with confidence interval showing lower and upper bound was used. The heterogeneity assessment was performed by obtaining the *I*^2^ statistic of the included studies, which measures their degree of inconsistency. The results of *I*^2^ were interpreted as follows: *I*^2^ < 25%—low or might not be important, 30–60%—moderate, 50–90%—substantial and 75–100% indicate a considerable heterogeneity. Subgroup analysis was performed based on geographic locations, study methodology, measurement side (left or right) and morphometric measurements to probe the sources of heterogeneity. Cochran’s *Q* was calculated as the weighted sum of squared differences and* p* < 0.10 was used as a cut-off for heterogeneity between the studies. The *I*^2^ statistic, weighted mean and standard error was calculated for each subgroup at 95% confidence intervals based on Cochrane guidelines [24].

### Quality assessment

The included studies were assessed for risk of bias using the Anatomical Quality Assessment (AQUA) tool to estimate its quality and reliability [25]. Studies were assessed based on five domains: objectives and subject characteristics, study design, methodology characterisation, descriptive anatomy, and reporting of study results. Each potential article source of bias was graded as low, high, and unclear as per AQUA guidelines. The included studies were independently assessed by two reviewers and the discrepancies were resolved by detailed discussion among the investigators.

## Results

### Study characteristics

**Fig. 2:**
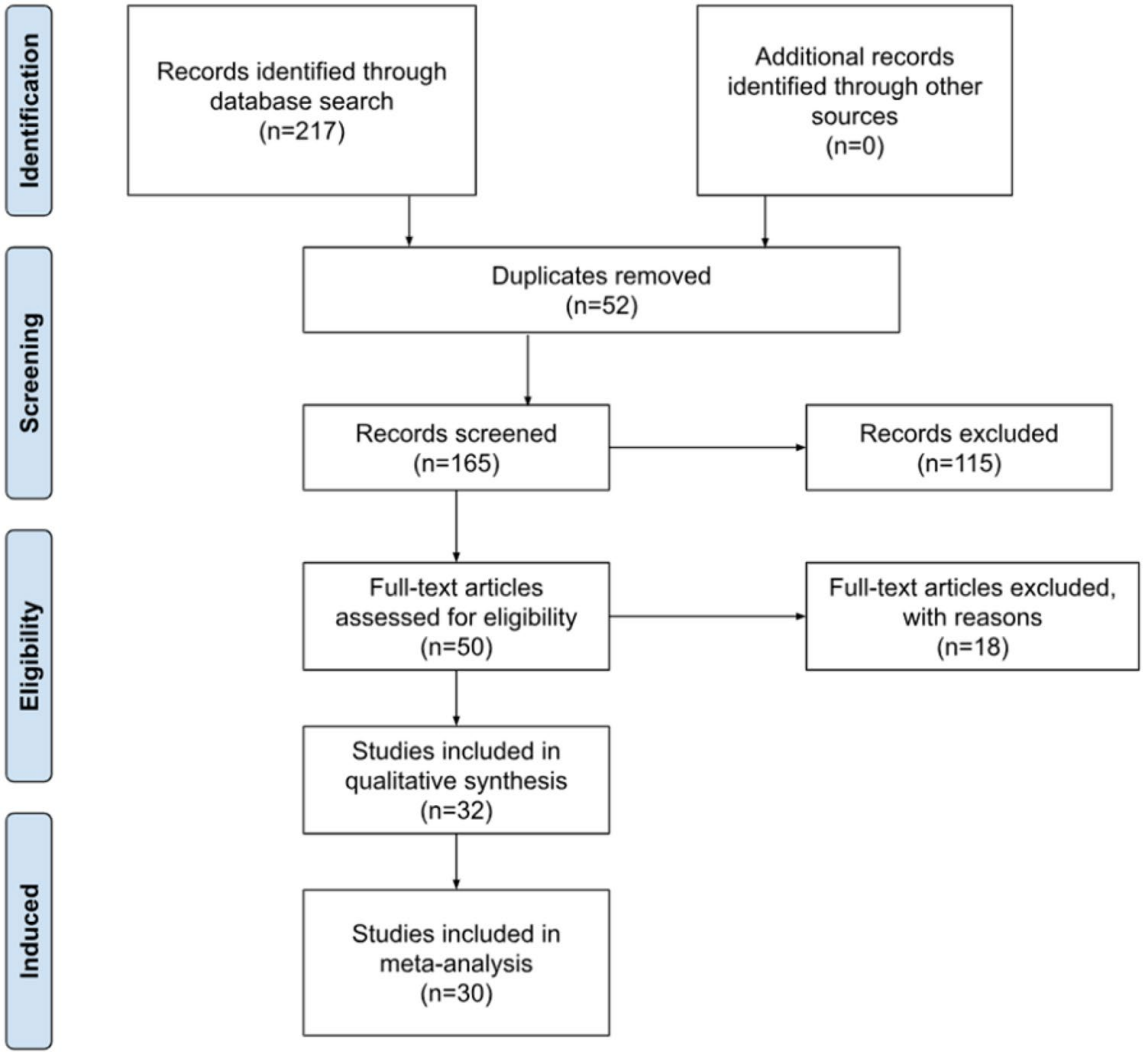
PRISMA flowchart of study selection

The PRISMA flowchart of study selection is given in Fig. 2. The initial search yielded 217 studies of which 52 were duplicates and 115 studies were irrelevant. Subsequently, 50 full text articles were assessed for eligibility of which 18 were excluded for different reasons. The 32 remaining articles which were included were ultrasonographic studies reporting the CSA of the sural nerve [10, 11, 26–55]. In total, there were 3193 sural nerves (healthy-2377; DM-816) among the 32 studies.

### Landmarks

The most common combination of external and anatomical landmarks used to assess the sural nerve were the lateral malleolus, ankle, and lesser saphenous vein [10, 11, 26–29, 31, 32, 34–36, 39–45, 48, 51, 52, 54, 55]. Half of studies measured the sural nerve at or just above the lateral malleolus, at the ankle or at the Achilles tendon [10, 26, 28, 29, 32, 34–36, 39, 41, 42, 44, 48, 51, 53, 55]. A smaller number of studies measured the sural nerve at distances ranging from 5 to 20 cm proximal to the lateral malleolus [11, 28, 31, 36, 40, 45, 47, 49]. Like the latter measurement site, some studies also defined their site of measurement as the mid-calf or between the heads of the gastrocnemius [28, 30, 33, 36, 45, 47, 50, 52]. However, some studies were vague in describing the site of measurement, with one only describing the site as adjacent to the lesser saphenous vein [27].

### Geographical location

**Fig. 3:**
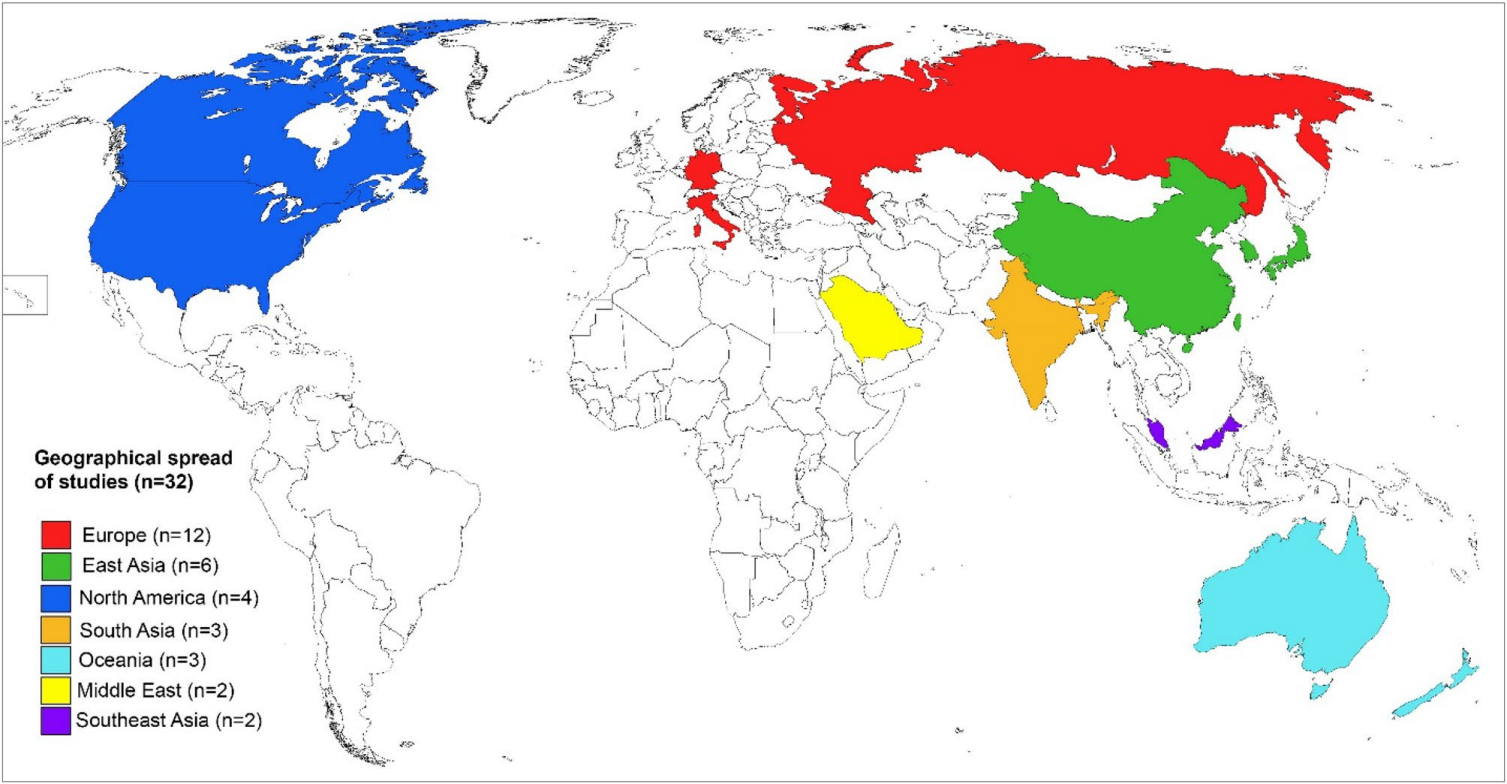
World map of countries from which included studies originate coloured by region. Countries from which at least one study was included are coloured based on the continent. A key to the left side includes the number of studies included for each continent

**Fig. 4:**
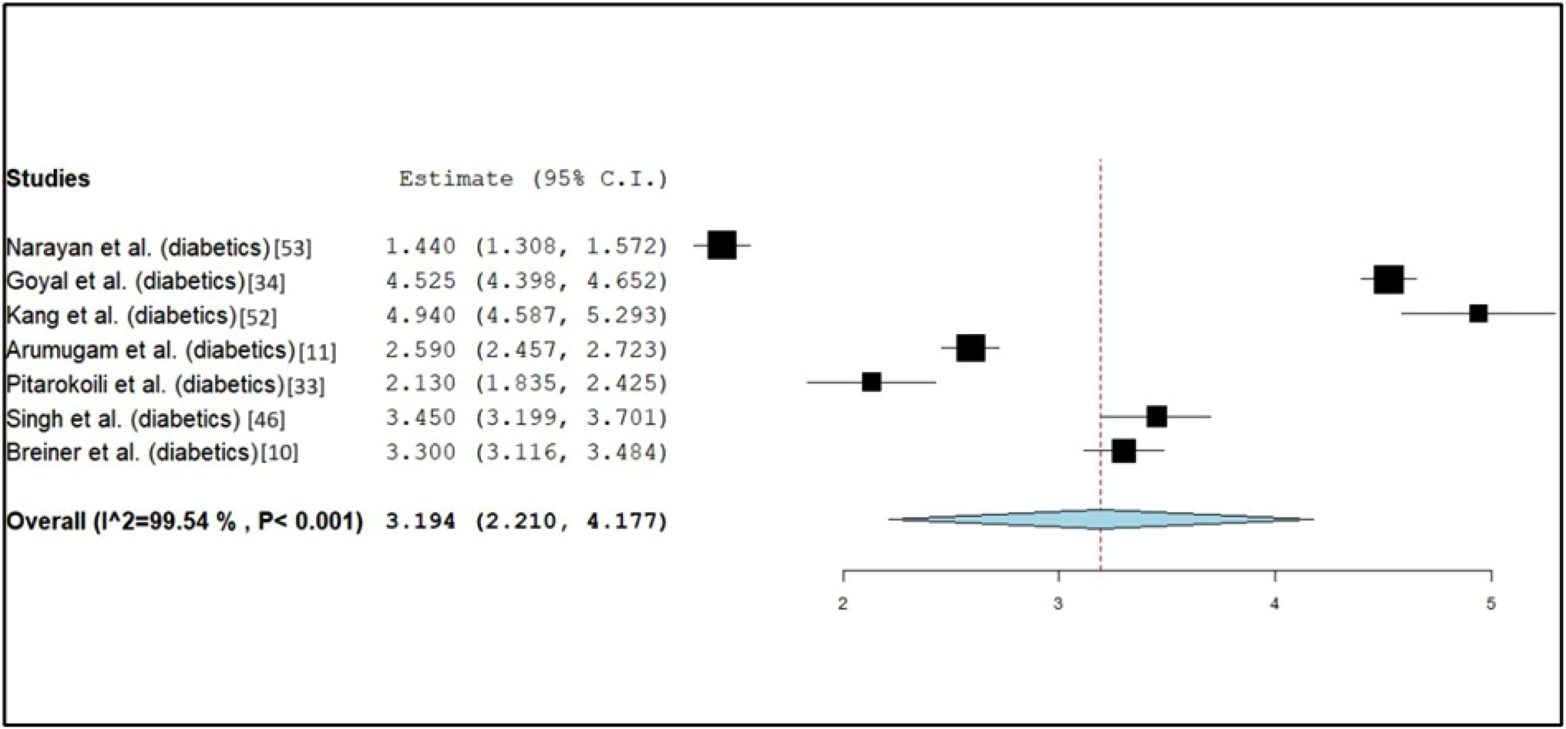
Forrest plot of reported estimate CSA of diabetic subjects and CI at 95% in studies that reported CSA of sural nerves in both healthy and diabetic subjects. Values can be found in Table 2a

The studies conducted at various geographical locations were summarised in Fig. 3. There were 12 studies contributing 870 sural nerves conducted in Europe [28, 29, 33, 36, 39, 43, 45, 47, 48, 50, 54, 55], 6 contributing 504 sural nerves in East Asia [26, 30, 31, 42, 49, 52], 4 contributing 435 sural nerves in North America [10, 32, 35, 41], 3 contributing 736 sural nerves in South Asia [34, 46, 53], 3 contributing 27 sural nerves in Oceania [37, 51], 2 contributing 447 sural nerves in Southeast Asia [11, 40] and 2 contributing 174 sural nerves in the Middle East [27, 44]. Seven of the European studies which contributed 438 sural nerves were conducted in Germany [28, 33, 36, 43, 47, 48, 50]. The studies from East Asia included significant representations from most countries including the People’s Republic of China [26, 31], South Korea [42, 52], Japan [30] and Taiwan [49]. In contrast, the Southeast Asian studies only drew participants from Malaysia, though one study recruited equal proportions of the three largest local ethnicities (Malays, Chinese and Indians) [40]. Participants in North America came from Canada and the United States only, and no studies recruited participants in South America or Africa (see Fig. 4).

### Quality assessment using AQUA tool

**Fig. 5:**
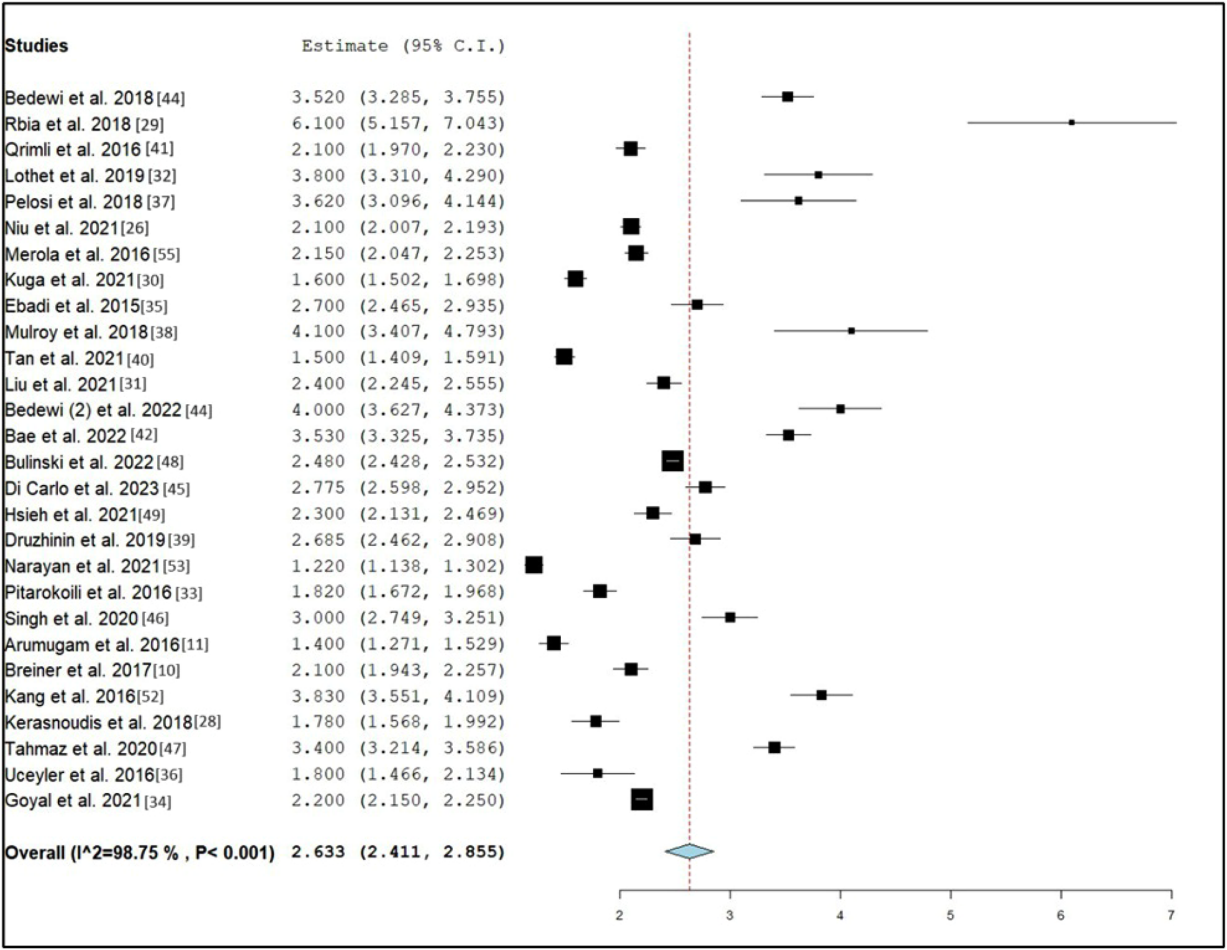
Forrest plot of reported estimate CSA of healthy subjects and CI at 95% in studies that reported CSA of sural nerves in healthy subjects only. Values can be found in Table 2b

Our AQUA assessment revealed a “low” risk of bias across all five domains for most studies (Table 1). In domain 1, five articles had a “high” or “unclear” risk of bias [26, 32, 34, 44, 48]. In domains 2, 3 and 4, a “high” or “unclear” risk of bias was observed in one study each [28, 32, 54]. In domain 5, four studies had a “high” or “unclear” risk of bias [28, 33, 34, 41] (see Fig. 5).

**Table 1:** Qualitative Assessment (QA): Risk of Bias, AQUA Tool

Author, Year	Domain 1	Domain 2	Domain 3	Domain 4	Domain 5
Breiner et al*.* (2017) [10]	Low	Low	Low	Low	Low
Arumugam et al*.* (2016) [11]	Low	Low	Low	Low	Low
Niu et al*.* (2021) [26]	unclear	Low	Low	Low	Low
Bedewi et al*.* (2018) [27]	Low	Low	Low	Low	Low
Kerasnoudis et al*.* (2018) [28]	Low	High	Low	Low	High
Rbia et al*.* (2018) [29]	Low	Low	Low	Low	Low
Kuga et al*.* (2021) [30]	Low	Low	Low	Low	Low
Liu et al*.* (2021) [31]	Low	Low	Low	Low	Low
Lothet et al*.* (2019) [32]	High	Low	High	Low	Low
Pitarokoili et al*.* (2016) [33]	Low	Low	Low	Low	unclear
Goyal et al*.* (2021) [34]	High	Low	Low	Low	unclear
Ebadi et al*.* (2015) [35]	Low	Low	Low	Low	Low
Üçeyler et al*.* (2016) [36]	Low	Low	Low	Low	Low
Pelosi et al*.* (2018) [37]	Low	Low	Low	Low	Low
Mulroy et al*.* (2018) [38]	Low	Low	Low	Low	Low
Druzhinin et al*.* (2019) [39]	Low	Low	Low	Low	Low
Tan et al*.* (2021) [40]	Low	Low	Low	Low	Low
Qrimli et al*.* (2016) [41]	Low	Low	Low	Low	Unclear
Bae et al*.* (2022) [42]	Low	Low	Low	Low	Low
Schubert et al*.* (2020) [43]	Low	Low	Low	Low	Low
Bedewi et al*.* (2022) [44]	unclear	Low	Low	Low	Low
Di Carlo et al*.* (2023) [45]	Low	Low	Low	Low	Low
Singh et al*.* (2020) [46]	Low	Low	Low	Low	Low
Tahmaz et al*.* (2020) [47]	Low	Low	Low	Low	Low
Bulinksi et al*.* (2022) [48]	High	Low	Low	Low	Low
Hsieh et al*.* (2021) [49]	Low	Low	Low	Low	Low
Grimm et al*.* (2016) [50]	Low	Low	Low	Low	Low
Hobbelink et al*.* (2018) [51]	Low	Low	Low	Low	Low
Kang et al*.* (2016) [52]	Low	Low	Low	Low	Low
Narayan et al*.* (2021) [53]	Low	Low	Low	Low	Low
Podnar et al*.* (2017) [54]	Low	Low	Low	unclear	Low
Merola et al*.* (2016) [55]	Low	Low	Low	Low	Low

Each domain comprises an aspect of anatomical research quality; domain 1 includes objective(s) and study characteristics, domain 2 includes study design, domain 3 includes methodology characterization, domain 4 includes descriptive anatomy and domain 5 includes reporting of results. Every study is rated based on their risk of not fulfilling good research practices; “low” suggests good practices with most/all aspects of the research domain addressed, “high” suggests aspects of the research domain unaddressed and “unclear” suggests uncertainty in whether such aspects of the research domain are addressed

Some of these biases included a small sample size, different sex ratios in different intra-study groups and study observations that did not fully answer the study questions. However, most of these biases were not relevant considerations for our meta-analysis, which collates as many samples as possible, regardless of demographic factors such as sex or study factors such as sample size. Moreover, some biases were inevitable, such as the lack of blinding of physicians performing ultrasonography on patients with observable symptoms. Therefore, none of the eligible studies were excluded based on biasness.

### Cross sectional area (CSA) of the sural nerve

The observations of the CSA are summarized in Table 2 and discussed in our meta-analysis. There were 7 studies that reported significant differences in the CSA of the sural nerve between healthy subjects and patients with DM [10, 11, 33, 34, 46, 52, 53]. Two studies reported significantly different CSAs of the sural nerves on both right and left sides in healthy and DM adults [34, 46].

**Table 2:** Summary of cross-sectional areas (CSA) of the sural nerve reported within included studies

Author (year)	Age	Cross-sectional area			
		Number of subjects	Number of sural nerves	Healthy (mm^2^)	Diabetic (mm^2^)
a: Studies that reported the CSA of sural nerves in healthy and diabetic subjects					
Breiner et al*.* (2017) [10]	44.1 ± 18.4	100	100	2.1 ± 0.8	
	46.6 ± 14.5	30	30		3.3 ± 1.0^a^*
	63.3 ± 12.5	67	67		3.3 ± 0.9^b^*
Arumugam et al*.* (2016) [11]	57.8	40	80	1.40 ± 0.59	
	59.1	100	199		2.59 ± 0.96 *
Pitarokoili et al*.* (2016) [33]	64.1 ± 11.2	55	55	1.82 ± 0.56	
	69.1 ± 12.17	44	44		2.13 ± 1.0^e^*
Goyal et al*.* (2021) [34]	44.2 ± 8.1	70	140	2.21 ± 0.32	
	48.2 ± 10.5	70	140		3.73 ± 0.77*
	50.3 ± 11.6	70	140		5.39 ± 0.62*
Singh et al*.* (2020) [46]	52.0 ± 11.8	30	30	3.0 ± 1.0^c^	
			30	3.0 ± 1.0^d^	
	55.1 ± 12.6	30	30		3.4 ± 1.0^c^*
			30		3.5 ± 1.0^d^*
Kang et al*.* (2016) [52]	65.0 ± 9.83	20	40	3.83 ± 0.9^ l^	
	66.2 ± 8.33	20	40		4.94 ± 1.14 l*
Narayan et al*.* (2021) [53]	56.0 ± 11	50	100	1.22 ± 0.42	
	53.7 ± 9.88	48	96		1.44 ± 0.66*
b: Studies that reported the CSA of the sural nerve in healthy subjects					
Niu et al*.* (2021) [26]	41.7 ± 15.8	111	111	2.1 ± 0.5	
Bedewi et al*.* (2018) [27]	38.3 ± 12.1	69	138	3.52 ± 1.41	
Kerasnoudis et al*.* (2018) [28]	49.3 ± 12.3	23	46	1.84 (0.52–3.1)^i^	
			46	1.73 (0.48–2.8)^j^	
			46	1.78 (0.56–2.9)^k^	
Rbia et al*.* (2018) [29]	59 ± 14	14	14	6.1 ± 1.8	
Kuga et al*.* (2021) [30]	44.2	100	100	1.6 ± 0.5	
Liu et al*.* (2021) [31]	50.08 ± 12.8	40	40	2.4 ± 0.5	
Lothet et al*.* (2019) [32]	20.0 ± 1.7	15	30	3.8 ± 1.37	
Ebadi et al*.* (2015) [35]	56.4 ± 15.7	25	25	2.7 ± 0.6	
Üçeyler et al*.* (2016) [36]	70 (39–84)	26	26	1.91 ± 0.87	
			26	1.69 ± 0.87	
Pelosi et al*.* (2018) [37]	67.1	14	14	3.62 ± 1.0	
Mulroy et al*.* (2018) [38]	48.6 ± 18.0	8	8	4.1 ± 1.0	
Tan et al*.* (2021) [40]	40.0 ± 14.4	84	168	1.5 ± 0.6	
Qrimli et al*.* (2016) [41]	44.2 ± 19.3 (M) /44.1 ± 18.4 (F)	100 (M = 30; F = 70)	96	2.1 ± 0.8^c^	
			87	2.1 ± 1^d^	
Bae et al*.* (2022) [42]	46.29 ± 14.19	107	107	3.77 ± 1.15	
			107	3.29 ± 1.01	
Bedewi et al*.* (2022) [44]	32.67 ± 7.07	18	18	4.0 ± 1.0^c^	
	32.67 ± 7.07	18	18	4.0 ± 1.3^d^	
Di Carlo et al*.* (2023) [45]	Unprovided	20	20	2.70 ± 0.57^c^	
			20	2.85 ± 0.58^d^	
Tahmaz et al*.* (2020) [47]	56.71 ± 22.8	80	160	3.4 ± 1.2	
Bulinski et al*.* (2022) [48]	75 ± 3	7	14	2.48 ± 0.10	
Hsieh et al*.* (2021) [49]	42.1 ± 14.0	66	66	2.3 ± 0.7	
Grimm et al*.* (2016) [50]	65.5 (24–78)	21	21	2 (1–3)	
Podnar et al*.* (2017) [54]	31	50	50	2 (1, 4)	
Merola et al*.* (2016) [55]	58.4 ± 16.1	70	140	2.15 ± 0.62	
c: Studies that reported the CSA of the sural nerve in paediatric subjects					
Druzhinin et al*.* (2019) [39]	3.27 (2–4)	11	11	1.64 ± 0.54^c^	
			11	1.67 ± 0.52^d^	
	5.61 (5–7)	15	15	1.80 ± 0.63^c^	
			15	1.93 ± 0.64^d^	
	8.47 (8–10)	10	10	2.13 ± 0.79^c^	
			10	2.15 ± 0.68^d^	
	11.3 (11–13)	7	7	2.15 ± 1.21^c^	
			7	2.22 ± 1.05^d^	
	14.8 (14–16)	7	7	2.64 ± 1.13^c^	
			7	2.73 ± 1.21^d^	
	24.5 (17–30)	22	22	2.73 ± 0.87^c^	
			22	2.64 ± 0.64^d^	
Schubert et al*.* (2020) [43]	2.84 (2–4)	58	58	1.1 ± 0.31^c^	
	5.72 (5–7)	58	58	1.26 ± 0.44^c^	
Hobbelink et al*.* (2018) [51]	8.2 ± 4.0	5	5	1.4 ± 0.2	

^a^T1DM

^b^T2DM

^c^Measurements were made on right side

^d^Measurements were made on left side

^e^Landmark of measurement: between medial and lateral heads of gastrocnemius

^f^Combined results from both subjects with DPN and subjects without DPN

^g^Landmark of measurement: thickness at the ankle

^h^Landmark of measurement: thickness at the leg

^i^Landmark of measurement: 14 cm proximal to malleolus externus

^j^Landmark of measurement: 7 cm proximal to malleolus externus

^k^Landmark of measurement: at the malleolus externus

^l^ ombined results from both left and right sides

Information of the mean age and age range of participants in each study, their number and cross-sectional area is provided. 2a. Information for studies reporting on both healthy and diabetic adults. 2b. Information for studies reporting on only healthy adults. 2c. Information for studies including paediatric (< 17 years of age) subjects

There were 25 studies that reported the CSA in healthy individuals with the mean CSA of SN ranged between 1.5 ± 0.6 and 6.1 ± 1.8 mm^2^ (Table 2b) [26–32, 35–49, 51, 54, 55]. Three studies included data of sural nerve CSAs in the paediatric population [39, 43, 51], with one of these studies stratifying across various age ranges from 2 to 30 years [39]. One of these studies reported an increasing CSA in successive paediatric age groups (Table 2c) [43].

No studies reported significant differences between CSA measurements performed on the right and left sural nerves regardless of the landmark used to assess the sural nerve or whether the subject was healthy or diabetic.

### Meta-analysis

#### Overall analysis: by diabetes mellitus type and by presence of diabetic polyneuropathy

A total of 32 studies reported sural nerve cross-sectional areas based on ultrasonography measurements [10, 11, 26–55]. Two were excluded from the meta-analysis due to a lack of data describing the standard deviations of sural nerve CSAs [50, 54]. Ultrasonography measurements of sural nerve CSAs calculated from 2085 healthy individuals aged 17 and above from 28 studies showed that the overall pooled weight mean CSA [upper bound, lower bound at 95% confidence interval (CI)] was 2.63 mm^2^ (CI [2.41, 2.85]) [10, 11, 26–38, 40–42, 44–55]. A separate meta-analysis of three studies reporting the sural nerve CSA in the healthy paediatric population (age range 2–10 years) is reported in the paediatric category [39, 43, 51].

Similarly, the weighted mean CSA from seven studies measuring 816 diabetic nerves was 3.19 mm^2^ (CI [2.21, 4.18]) [10, 11, 33, 34, 46, 52, 53]. Of this, one study reported type 1 diabetic sural nerves with a mean CSA of 3.30 mm^2^ (CI [2.94, 3.66]) [10] and all seven studies reported type 2 diabetic populations with a sural nerve mean CSA of 3.19 mm^2^ (CI [2.19, 4.20]) [10, 11, 33, 34, 46, 52, 53]. Two studies that evaluated sural nerve CSAs from a total of 236 type 2 diabetic patients without DPN yielded a mean CSA of 2.57 mm^2^ (CI [0.36, 4.79]) [34, 53] while three studies which evaluated 379 type 2 diabetic patients with DPN yielded a mean CSA of 4.29 mm^2^ (CI [2.23, 6.35]) [11, 34, 52].

The mean sural nerve CSA was largest in type 1 diabetics in comparison with type 2 diabetics and the healthy population. There was a statistically insignificant increase in the weighted sural nerve mean CSAs when comparing either type 1 or type 2 diabetic populations as well as non-DPN diabetics with healthy individuals (Table 3a). However, DPN patients showed a statistically significant increase in sural nerve CSA when compared to healthy individuals. A considerable heterogeneity (*I*^2^ > 95%) with Cochrane *Q* statistic of P < 0.001 was observed, indicating variation among the studies (Table 3).

**Table 3:** Summary of pooled weighted mean sural nerve CSAs based on healthy and diabetic populations

Subgroup analysis	Study references	Number of studies included	Number of sural nerves evaluated	Weighted mean CSA (mm^2^)	CI at 95%	Standard error	*I*^2^ (%)
a: Pooled weighted mean sural nerve CSAs in healthy and diabetic adults							
Healthy adults	[10, 11, 26–38, 40–42, 44–55]	28	2085	2.63	[2.41, 2.85]	0.113	98.8
Diabetic adults	[10, 11, 33, 34, 46, 52, 53]	7	816	3.19	[2.21, 4.18]	0.502	99.5
Type I diabetic adults	[10]	1	30	3.30	[2.94, 3.66]	0.183	^
Type II diabetic adults	[10, 11, 33, 34, 46, 52, 53]	7	786	3.19	[2.19, 4.20]	0.512	99.5
Diabetic, non-DPN adults	[34, 53]	2	236	2.57	[0.36, 4.79]	1.13	99.8
Diabetic, DPN adults	[11, 34, 52]	3	379	4.29	[2.23, 6.35]	1.05	99.8
Healthy adults (only from studies including diabetics)	[10, 11, 33, 34, 46, 52, 53]	7	575	2.21	[1.74, 2.69]	0.242	99.2
b: Pooled weighted mean sural nerve CSAs in adults by geographical region							
Healthy adults (Europe)	[28, 29, 33, 36, 39, 45, 47, 48, 55]	9	539	2.59	[2.27, 2.91]	0.164	97.6
Diabetic adults (Europe)	[33]	1	44	2.13	[1.83, 2.43]	0.151	^
Healthy adults (East Asia)	[26, 30, 31, 42, 49, 52]	6	464	2.62	[2.07, 3.17]	0.280	98.9
Diabetic adults (East Asia)	[52]	1	40	4.94	[4.59, 5.29]	0.180	^
Healthy adults (North America)	[10, 32, 35, 41]	4	338	2.61	[2.15, 3.06]	0.232	95.1
Diabetic adults (North America)	[10]	1	97	3.3	[3.12, 3.48]	0.0941	^
Healthy adults (South Asia)	[34, 46, 53]	3	300	2.13	[1.33, 2.94]	0.411	99.6
Diabetic adults (South Asia)	[34, 46, 53]	3	436	3.14	[1.01, 5.27]	1.09	99.8
Diabetic, non-DPN adults (South Asia)	[34, 53]	2	236	2.57	[0.355, 4.79]	1.13	99.8
Diabetic, DPN adults (South Asia)	[34]	1	140	5.35	[5.25, 5.45]	0.0507	^
Healthy adults (Southeast Asia)	[11, 40]	2	248	1.46	[1.37, 1.56]	0.0490	35.1
Diabetic adults (Southeast Asia)	[11]	1	199	2.59	[2.46, 2.72]	0.0681	^
Healthy adults (Oceania)	[37]	1	22	3.80	[3.34, 4.26]	0.233	^
Healthy adults (Middle East)	[27, 44]	2	174	3.74	[3.27, 4.21]	0.239	78.0
c: Pooled weighted mean sural nerve CSAs in adults by measurement site							
Proximal to lateral malleolus or ankle	[10, 26–29, 32, 34–36, 39, 41, 42, 44, 48, 53, 55]	16	1254	2.68	[2.40, 2.95]	0.140	98.8
Distal calf; 7–10 cm proximal to lateral malleolus	[11, 28, 31, 37, 38, 40, 42, 46, 49]	9	589	2.55	[2.06, 3.04]	0.250	98.5
Mid-calf; > 10 cm from lateral malleolus	[28, 30, 33, 36, 45, 47, 52]	7	467	2.42	[1.80, 3.04]	0.318	98.8
d: Pooled weighted mean sural nerve CSAs in adults by measurement side							
Right	[34, 39, 41, 44–46]	6	437	2.94	[2.30, 3.58]	0.326	97.6
Right (with Breiner et al*.*)	[34, 39, 41, 43–46]	7	534	2.93	[2.45, 3.41]	0.246	96.9
Left	[34, 39, 41, 44–46]	6	437	2.98	[2.35, 3.60]	0.319	97.4
Left (with Schubert et al*.*)	[10, 34, 39, 41, 44–46]	7	553	2.70	[1.86, 3.53]	0.428	99.3
e: Pooled weighted mean sural nerve CSAs in paediatric and adult populations							
Children (< 11 years)	[39, 43]	2	188	1.53	[0.842, 2.21]	0.349	98.6
Adolescence (11–16 years)	[39]	1	28	2.44	[2.02, 2.85]	0.211	^
Adult (> 16 years)	[10, 11, 26–38, 40–42, 44–55]	28	2085	2.63	[2.41, 2.85]	0.113	98.8

^Where only one study fulfils the criteria, its mean is reported and no *I*^2^ value is calculated

Each row provides the results of the Student’s T-test performed to compare two pooled groups of studies meeting various inclusion criteria (such as comparisons between diabetic and non-diabetic adults), including the number of sural nerves pooled, the weighted mean, its 95% confidence interval, the standard error and the I2 statistic. Whenever one group contained only one study which met the inclusion criterion, no I2 statistic was provided. 3a. Comparisons between healthy and diabetic adults with and without diabetic polyneuropathy. 3b. Comparisons between diabetic and healthy adults by continent. 3c. Comparisons between measurements made at different sites along the sural nerve. 3d. Comparisons between measurements made on left and right feet. 3e. Comparisons between healthy adults and the healthy paediatric population

Subgroup analysis: Meta analysis on the data of sural nerve CSA were summarised in Table 3b, 3c and 3d. Overall, there is a variation in the CSA values with reference to geographical region, measurement sites and sides, age, height, weight, and BMI.

Some variation in the data of sural nerve CSA was observed when comparing by geographical region, measurement sites and sides [10, 28, 34, 36, 39, 41–46], age and height, weight, and BMI [32]. (Table 3b–d).

Region-specific analysis comparing healthy and diabetic populations revealed significant differences in sural nerve CSAs within articles involving East Asian participants (*p* < 0.0154, difference: 2.32, CI [0.445, 4.20]) and Southeast Asian participants (*p* < 0.0001, difference: 1.13, CI [0.969, 1.29]), but not for other regions. However, it is worth noting that the diabetic cohorts of both regions were limited to single studies each that included only diabetics with diagnosed DPN. Additionally, a significant difference between diabetics diagnosed with DPN and healthy participants from South Asian studies (*p* < 0.0001, difference: 3.22, CI [2.03, 4.40]).The mean CSAs of the sural nerve in healthy individuals varied greatly by region but were particularly low in Southeast Asia (1.46 mm^2^, CI [1.37, 1.56]) and high in the Middle East (3.74 mm^2^, CI [3.27, 4.21]) as well as Oceania (3.80 mm^2^, CI [3.34, 4.26]), though the latter had a relatively small sample size (Table 3b).

There were no significant differences in sural nerve CSAs between the three categories based on measurement sites i.e., (I) at or just above the lateral malleolus/ankle up to 5 cm away, (II) above the lateral malleolus/ankle, from 5 to 10 cm away, and (III) near the mid-calf, > 10 cm away from the lateral malleolus. (Table 3c). There was no significant difference in sural nerve CSAs between left or right lower limbs when accounting only for studies which included both left and right measurements (*p* = 0.942, difference: – 0.033, CI [– 0.929, 0.863]) or when including all 8 studies (*p* = 0.623, difference: – 0.234, CI [– 1.17, 0.700]).

There was a significant increase in sural nerve CSA when comparing children with adults (*p* = 0.0047, difference: 1.10, CI [0.337, 1.87]), but no statistically meaningful difference comparing children with adolescence or other permutations of the three age groups (Table 3e). Based on the drastic increase in mean CSA from children to adolescence and minimal change from adolescence to adulthood, we surmise that the increase in sural nerve CSA might occur during late childhood and/or early adolescence and that the data failed to reflect due to a small sample size in our adolescence group (*n* = 28) as compared with the adult group (*n* = 2085).

One study which examined the sural nerve CSAs of tall and heavy individuals separately did not identify a statistically significant difference in the CSAs when compared with a large pool of controls, though the mean sural nerve CSA of their control data differed significantly from the other studies included in this systematic review [32].

## Discussion

Diabetic polyneuropathy is associated with an increased sural nerve CSA which varies across geographical region.

As a sensory peripheral nerve that is well-associated with diabetic neuropathies, measurements of the sural nerve have the potential to be incorporated in clinical decision-making in diabetic patients. In this systematic review, we performed a meta-analysis of sural nerve CSAs from 31 studies to identify mean values among healthy and diabetic individuals, individuals from different regions, of different ages and BMIs, as well as across different measurement sites in the distal limb.

First, we demonstrate a significant range of sural nerve CSAs for both healthy and diabetic individuals. We did not notice any statistically meaningful difference in mean CSAs between healthy and diabetic adults in our overarching analysis, despite an increase in mean CSA in diabetic adults. This also applied to type I and type II diabetics when separately compared with healthy adults and agrees with the fact that diabetes mellitus is principally an endocrine disease that results in complications from chronically poor glycaemic control [8]. The extensive variance in population sural nerve CSAs suggests that it is unlikely to be feasibly employed in a clinical diagnostic setting, such as to distinguish diabetics from healthy individuals.

However, we noted a statistically meaningful difference in sural nerve CSAs when comparing diabetic adults with diabetic polyneuropathy to healthy adults, which was not present when comparing non-DPN diabetics to healthy adults. Many studies have reported changes to nerve conduction and sensory perception in DPN patients [4, 5, 13]. Our results underscore the hypertrophic state of the sural nerve in DPN patients and raise the possibility of the use of ultrasonography of nerve CSA as an additional diagnostic tool or criteria in DPN.

Our data also suggests that diabetes mellitus alone does not lead to any change in sural nerve CSA and that morphological analysis of peripheral nerves such as the sural nerve is unlikely to have any capacity in distinguishing diabetics from healthy adults. Nonetheless, we recognize that two studies separately demonstrated a statistically significant difference in non-DPN diabetic sural nerve CSAs from healthy adults, despite a meta-analysis of all studies from their region showing no such difference [34, 53]. We surmise this to be a consequence of the extensive variation of sural nerve CSAs from even different localities, given the lack of other selection biases in the recruitment process of participants from our investigation.

Second, given the significant increase in sural nerve CSA in DPN diabetics, ultrasonography of the sural nerve may be a useful method in gauging the development of DPN across time in diabetics. Future studies examining gradual changes in sural nerve morphology in diabetics, such as through routine follow-up, may inform about the relationship between changes in sural nerve morphology and DPN. Given the relatively cheap cost of ultrasonography and its frequent use by general practitioners, its deployment in community care settings is a potential option to address DPN progression [14].

The variation in sural nerve CSA based on location along the lower limb highlights individual-specific differences that may not be sufficiently large to necessitate consideration in forming reference values as well as the based on the side of measurement.

While suggestive that ultrasonography of the sural nerve on either leg are equally valid to obtain CSA values for diagnostics, it would be interesting to explore datasets which segregate measurements based on dominant and non-dominant foot. Such datasets would be integral in evaluating whether dominance modifies sural nerve CSA and thus, confirming whether side dominance is an important consideration for such diagnostic measurements.

The study which reported an inverse relationship had the oldest patients (mean: 56.7 years, range 18–98 years) among all the studies [47]. One other study that reported an absence of a correlation also calculated a mean sural nerve CSA which was smaller in adults over 60 years of age as compared with adults aged 40–59 or adults younger than 40 [42]. Although these values were not statistically different, they suggest that the elderly demographic is largely understudied in the context of peripheral nerve morphology and that an inverse correlation of the sural nerve CSA with age above 60 should be considered. Such a change would be expected in the aging population, where progressive physiological dysfunction that, at a cellular level, includes increased demyelination, axonal shrinkage and mitochondrial loss, can drive the degeneration of peripheral nervous tissue [56, 57]. Taking into consideration such changes in sural nerve CSA from aging will therefore be important in potential situations where sural nerve CSA is used to inform clinical decisions (such as lowering the cut-off value for a pathology).

There is lack of inverse correlation with reference to the data of CSA, height, weight and BMI, thus, it is possible that these body proportions are positively associated with sural nerve CSA to some limited extent.

Ultimately, future studies are needed to evaluate the relationships between body metrics and sural nerve CSA in healthy and diabetic populations. Such studies will likely require larger cohort sizes and need to consider the effects of confounding variables, such as age and body metrics. Additional studies will also be important to establish reference ranges for healthy sural nerve CSAs based on these measures and for different geographical regions.

### Study limitations

Our systematic review was limited in our ability to control the measurement techniques in our incorporated studies. While we did our best to ensure all studies followed standard protocol in our AQUA review of each study, we note that not all studies (i) practiced identical ultrasonographic techniques, (ii) utilized identical or standardized measurement sites on the distal leg, (iii) took measures to limit intra-observer variability, (iv) utilized similar equipment etcetera.

For example, many studies did not mention the specific frequency employed during ultrasonography and provided only the frequency range of their ultrasound probe. While most ranges overlap, we note that a significant number of studies used an 18 MHz frequency during ultrasonography while some other studies used probes which could not utilize this frequency. However, we did notice that all studies employed a similar methodology when measuring the nerve CSA—by tracing the inner hyperechoic rim on a transverse section of the nerve.

Secondly, the sample size for some studies was especially small, such as in the case of studies conducted in Oceania, where we also noted limited data heterogeneity. This prevented us from drawing conclusions that would be applicable for the region. Moreover, the inter- and intra-regional variability suggests that larger samples must be obtained to increase the accuracy of our sample as a representation of the population. A similar lack of sample size from studies that discuss the relationship of the sural nerve CSA with age, height, weight, and BMI limit our ability to draw conclusions on these.

Similarly, while our incorporated data represents many ethnicities, we lacked data from regions such as South America and Africa as well as large countries such as Mexico, Pakistan, and Brazil, limiting the utility of the findings in our subsequent meta-analyses for their local ethnicities.

We also highlight that the pooling of data for our meta-analysis introduces study bias when comparing various subgroups. For example, studies which provide only healthy data do not provide data on matched diabetics. This means the local diabetic populations of studies involving only healthy participants are not sampled within our pooled data for diabetic measurements, thereby leading to a biased comparison. We accounted for this by comparing healthy and diabetic adults from only studies incorporating both these demographics and noted a lower mean CSA for healthy adults among these studies compared with our pooled sural nerve CSA data for healthy adults, but also a similar lack of statistically significant difference in CSA values comparing healthy and diabetic adults.

### Usage of ultrasonography in diagnosing diabetic neuropathy

Presently, peripheral neuropathies are typically diagnosed based on a constellation of signs and symptoms recorded during history taking and physical examination, as well as quantitative testing, such as nerve conduction studies [58, 59]. Conversely, the usage of ultrasonography as an alternative tool, by CSA measurement, has been met with uncertainties to do with diagnostic value [60]. Differences in patient populations and anatomical variation contribute to variations in ideal diagnostic cut-off values, consequenting in unsatisfactory sensitivity and false negative rates [60]. Moreover, patient biometrics, such as age, weight, and BMI, are reported to correlate with nerve CSA, suggesting that a one-size-fits-all diagnostic cut-off to be inadequate [61, 62].

Nonetheless, our finding suggests that neuropathic changes associated with DM may manifest significantly in changes to the CSA of the sural nerve. In this regard, we propose that ultrasonography to detect variations in sural nerve CSAs may hold clinical usefulness as a complement to existing diagnostic modalities. Beekman et al. reported an increase in sensitivity and specificity of electrodiagnostic testing for ulnar nerve entrapment when ulnar nerve diameters, measured by high-resolution ultrasonography, were included as part of the diagnostic workup [63]. Region-specific or even hospital-specific diagnostic cut-offs may then be employed, given how our meta-analysis shows significant differences in sural nerve CSAs on a regional basis, to provide additional clinical evidence toward a pathological state of the peripheral nervous system, and interpreted based on the presence of appropriate signs and symptoms.

## Conclusion

The sural nerve is a peripheral nerve well-involved in polyneuropathies. Our study provides pooled and weighted mean CSAs based on different geographical locations, age, body metrics, anatomical sites and other parameters in healthy and DM patients that may act as references for clinicians conducting evaluations of the sural nerve. We show that the mean CSA of the sural nerve is significantly larger in DM patients with DPN across all regions and when pooled together, suggesting that sural nerve CSAs derived by USG could act as a clinical complement to existing diagnostic tools, such as nerve conduction studies. We show that an age-dependent increase in the CSA of healthy sural nerves occurs when comparing the paediatric population with adults. However, future studies are needed to elucidate the associations between body metrics and age with sural nerve CSA, as well as identify potential differences in mean CSA values from different geographical regions, which we found greatly varied even in healthy adults.

## Data Availability

All data generated or analysed during this study are included in this published article in the form of tables.
